# Standard operating protocol for utilizing energy‐based devices in aesthetic practice

**DOI:** 10.1111/jocd.16510

**Published:** 2024-09-04

**Authors:** Venkataram Mysore, M. Deepthi, B. S. Chandrashekar, Swapnil D. Shah, Michael H. Gold, S. R. Shivani, Pooja Kanumuru, P. Anirudh

**Affiliations:** ^1^ Venkat Center for Skin, ENT & Plastic Surgery Bangalore Karnataka India; ^2^ Consultant Dermatologist Ashraya Skin & Neuro Clinic Bangalore Karnataka India; ^3^ Department of Dermatology CUTIS Academy of Cutaneous Sciences Bengaluru Karnataka India; ^4^ Department of Dermatology Ashwini Rural Medical College Solapur Maharashtra India; ^5^ Gold Skin Care Center, Advanced Aesthetics Medical Spa The Laser and Rejuvenation Center, and Tennessee Clinical Research Center Nashville Tennessee USA; ^6^ Skin Lab Clinic Delhi India

**Keywords:** cryolipolysis, laser hair removal, laser safety, MnRF, Q‐switched lasers

## Abstract

**Background:**

Lasers and other energy‐based devices are increasingly becoming popular in aesthetic practice. Many centers employ doctors or technicians to perform these procedures where treating doctor and operating doctor may be different. Hence the need for standard operative protocols, to be followed while performing these procedures to avoid mistakes, complications and to get optimum results. In the current review article, group of doctors who have worked with these energy‐based devices over many years worked together and suggested the protocols to be followed for the most commonly used energy‐based procedures.

**Aim:**

To provide Standard operating protocols for the operator and staff to ensure, efficacy, safety, for the patient and for the devices.

**Methods:**

The following protocols have been drafted based on the best practices followed by the authors in their clinics and reflect their consensus opinion. The objective is to provide operating protocols in a standard format, which can be of use by practicing dermatologists and their staff. The protocols include both general guidelines for the laser room and specific protocols for different machines. The draft follows the following schema:

- General instructions for all the energy‐based devices.
- Specific protocols for different devices: Laser hair removal, fractional lasers, Q‐switched lasers, fractional microneedling radiofrequency and cryolipolysis.

**Conclusions:**

The protocols proposed help to maintain the uniformity and avoid complications. However, these instructions are generalized and not machine or lesion specific. There may be variations in the protocols depending on the treatment lesion and treating doctor as well as machine.

## 1 INTRODUCTION

Lasers and other energy‐based devices such as, intense pulsed light, radiofrequency form the cornerstone of aesthetic treatments. Aesthetic practice not only relies on providing effective and safe treatments customized to suit the requirements of the individual patient but also, at the same time on achieving a fine balance with hospitality and patient comfort. Most of these treatments are delivered through junior doctors, nurses, nurse practitioners and technicians. While doing so, it is important to ensure that the treatments are properly supervised and delivered as per standard protocols to ensure efficacy and safety, for the patient as well as for the devices.

The following protocols have been drafted based on the best practices followed by the authors in their clinics and reflect their consensus opinion. The objective is to provide operating protocols in a standard format, which can be of use by practicing dermatologists and their staff.

The protocols include both general guidelines for the laser room and specific protocols for different machines. The draft follows the following schema:

- General instructions for all the energy‐based devices.
- Specific protocols for different devices: Laser hair removal, fractional lasers, Q‐switched lasers, fractional microneedling radiofrequency and cryolipolysis.

## 2 GENERAL INSTRUCTIONS WHILE PERFORMING ENERGY BASED DEVICES

### 2.1 Pre‐procedure instructions to patients

1. Patient is advised not to undergo any facials, bleaching, waxing or any other cosmetic treatment for 1 week prior to the procedure (applicable for procedures involving face) and avoid waxing/threading 1 month before procedure.
2. Emphasize on the regular use of sunscreen, moisturizer, and depigmenting creams.
3. Ask to stop active creams 2 days prior to the procedure (especially topical retinoids, glycolic acid etc.).
4. Ask to avoid any travel plans, especially activities that involve lot of direct sunlight exposure 1 week before and after procedure.
5. Avoid procedures, especially ablative, in patients who have social events within a week or two.
6. Avoid procedures in case of any photodermatitis/ active infection/ eczema/extremely dry skin/tanned skin at the site of treatment.
7. In case of HSV infection, prophylactic antiviral coverage must be started 2 days prior to the treatment and continued 5–7 days post procedure (14 days for resurfacing procedures).^1^, ^2^
8. Take a detailed history of all the medicines/creams the patient is on including usage of herbal supplements, aromatic oils, and home‐based remedies.
9. Patient is advised about the use of topical anesthesia, if necessary, 30 to 45 min before treatment for procedures involving pain or those with low threshold for pain.^3^, ^4^, ^5^
10. Ensure that the staff/doctors will make the procedure very comfortable for the patient to minimize the pain.
11. Advise patients not to be anxious during the procedure as it will be a very comfortable procedure.
12. In case of CO2 lasers involving intense heat, caution the patient regarding the feel of mild to moderate heat sensation.
13. If any doubts regarding the treatment, the patients are advised to clarify with the nurses/doctors.
14. A simple pre procedure check list signed by patient can help in ruling out any contraindications.^16^

### 2.2 Instructions for staff handling lasers

1. Dress appropriately in a professional manner, as that first interaction with the patient sets the stage for everything that follows.
2. Greet the patient with a smile, introduce yourself to the patient in an appropriate manner preferably in their preferred language, build a rapport and make the patient comfortable. Ensure that the right patient has been shifted for the right procedure.
3. Ask the patient to share the reason for being at the clinic—do not assume.
4. Complete the pre procedure check list to rule out any contraindications (as mentioned in Table 1).
5. Make sure the patient fills the relevant consent form and the consent is signed separately before every repeated session with date. In case of minor take signature from the guardian. The consent should include consent for photography, specifically stating whether the patient agrees for the use of the photos for presentation/publication/website/social media.^6^
6. Separate consent form needs to be taken for anesthesia (local/topical) if needed.
7. Take appropriate photos against blue or black background screens. Always take photographs in the same room, if possible, with the same camera, same angles, and lightings. Preferably take front, side and 45 degrees profile views for facial procedures. Close‐up pics can be taken wherever applicable. Scars should be photographed at a tangent angle. Transfer the images to the software immediately. Review the photos and discard the ones which are not clear.^7^, ^8^
8. Ensure adequate lighting in the procedure room.
9. Keep the air conditioning/ventilation at an ambient level to ensure maximum comfort to the patient.
10. Ensure smoke evacuator is in the room for the procedures where a plume will be produced (carbon peel, RF, ablative lasers).^9^
11. Ensure cooling devices like Zimmer are switched on and ice packs wherever necessary is available.^10^
12. Ask for any history of parlor procedures such as waxing, threading, bleaching or permanent makeup procedures in the areas to be treated.
13. Careful examination of the skin to be done prior to starting the procedure for any active infection, eczema, dryness, redness. In case of any doubt opinion from the treatment doctor should be taken.
14. Apply headband to push hair back in relevant cases.
15. Cleanse the treatment area with a facewash or a cleanser. Remove makeup with micellar water or an appropriate cleanser using cotton swabs or soft gauze.
16. Protect the areas, like eyebrows, tattoos, moles with opaque sticker or micro pore, which do not require treatment to prevent laser light from lasing them.
17. Ensure safety goggles have been put for patient, operator, and other personnel, appropriate for the device used.
18. Use only white markers pencils/pens to mark the area/lesion. Avoid using colored pens.
19. Once EMLA is applied, watch for skin reactions [intense erythema, burning] every 15 min and if observed inform the doctor immediately.
20. Usage of disposable ice packs is advised for procedures that may involve exudation of blood and serum such as fractional laser to avoid cross contamination.
21. Do not recommend any skin care products other than what is recommended by the protocol or the treating doctor.
22. Do not discuss doubts/share any experience of side effects or complications in front of the patient. Use discretion during communications with the staff in patient's presence.

**TABLE 1:** Pre procedure questionnaire (Check list for Aesthetic procedures).

S.NO	Questionnaire
1.	What is the nature of your work?
2.	Do you have important meeting/functions in the coming 1 week?
3.	Are you allergic to any medication or ointments?
4.	Are you sensitive to light? (i. e., do you have itching, burning sensation, redness while going out in sunlight?)
5.	Have you had an episode of herpes (blisters over lips)?
6.	Have you had anesthesia before?
7.	Do you suffer from an infection or inflammation that you are aware of if so, what?
8.	Are you pregnant?
9.	Are you breast feeding?
10.	Do you have tendency to scar, pigment, Keloid, vitiligo, psoriasis?
11.	Have you undergone facials, bleaching or any other chemical treatment in this week?
12.	Are you on painkillers, Vitamin E, herbal medicines, anticoagulants?
13.	Do you have any systemic illnesses?
14.	Are you on treatment for any health issue, if so what treatment? Do you use sunscreen?
15.	Do you use sunscreen?

### 2.3 General instructions for doctors

1. See the patient on arrival before topical anesthesia is applied, introduce yourself and talk to the patient, establish rapport.
2. Read notes, refer previous treatments, assess the results of previous treatment, enter these in the appropriate form, check / ask the questions in pre procedure checklist. Inform the patient in case of any change in the procedure.
3. Check for the ongoing medications and any recent skin allergies.
4. Ensure that the pre procedure photographs are adequate.
5. Hand over the patient to the staff for documentation, check the consent, photos, sign the consent as treating doctor.
6. Perform any other specialized photography or imaging technique whenever necessary.
7. Use the time for the topical anesthesia cream to plan the procedure and the parameters and making sure that the device is in working order, and make the patient at ease.
8. Wherever necessary regional blocks should be given (Perioral wrinkles fractional resurfacing).
9. Once the topical anesthesia patch time is up, the cream has to be removed with cotton and the area is cleaned and check the skin for any redness/ blisters at the site of topical anesthesia patch.
10. Make sure appropriate protection glasses are worn by all personnel in the room.
11. Check whether Cooling device is already switched on and set the appropriate settings for non contact cooling device by checking on operator skin first. Avoid using the Cooling device abruptly on the patient's skin without informing the patient as it can be intimidating.
12. Patient can be asked to test the coldness of air before the procedure and post procedure. For initiative patients, they can be asked to hold the Cooling device by themselves during the procedure.
13. While performing the procedure, constantly communicate with the patient. Ask for any discomfort, pain or burning sensation. In selected cases, as per discretion of the treating physician, a test spot may need to be performed to see if the appropriate settings have been chosen and that there are no other untoward effects moving forward with the procedure.
14. After the procedure is completed, the skin is cooled using cold packs (in necessary procedures). Talk to the patient, take/check photos post procedure, give post procedure instructions, clear any doubts that the patient has, enter the final parameters, enter the treatment plan, check if any additional drugs are needed for the primary condition.
15. Any untoward reaction should be documented in appropriate forms and sign the treatment chart.
16. Issue post procedure prescription and any special instructions and then hand over the patient to staff after mentioning post procedure instructions multiple times. If the patient has to be followed up specially (ablative procedures and procedures done on weekend), instruct the reception staff. The instructions should specifically mention a telephone number for contact in case of any emergency.
17. Ensure that the staff/ treating doctor follows the patient with a telephone call the evening of the procedure and next day, followed by on 3rd and 7th day for procedures with prolonged downtime. Example: Laser resurfacing.
18. A “what to do if something goes wrong” sheet should be easily available in all procedure rooms.
19. An experienced laser consultant should be available for supervising the procedures regarding any device, parameters issues or any complications.
20. All the procedure parameters informing about handpieces can be printed and posted on Laser room cupboards for reference.

### 2.4 General sops for handling energy based devices

#### 2.4.1 General measures for Laser room

1. To be extremely mindful while walking in a room with lasers, as accidental slippage over the machine and its parts may cause catastrophic damages both to machine and manpower leading to absenteeism and financial losses.
2. To be mindful of the foot switch and wires on the floor.
3. To wear Laser room slippers or appropriate footwear before entering the Laser room.
4. Avoid trimming of hairs in the laser rooms.
5. Keep the windows and doors closed in the laser room to avoid dust accumulating.
6. Vacuum clean the laser room every day to avoid dust and hair settling over the laser lens, especially where melanin/black color is the chromophore.
7. Avoid inflammable substances around the laser and avoid alcohol swabs for cleansing the treatment area instead use poviodine solution.
8. Have fire extinguisher immediately outside the laser room in case of an emergency.
9. Air conditioning should be switched on 30 min prior to the procedure.
10. A Laser sign board/ light has to be displayed on operating doors while Laser operation is going on.^11^, ^12^, ^13^

#### 2.4.2 Handling the arm (handpiece) of the laser

1. The arm of the device (hand piece) should be gently removed from its socket using perpendicular pressure to avoid damage to the cables/lens and in difficult situations take the help of senior nursing staff.
2. Avoid holding other things in the hand and do not pull or push the machine by holding arm.
3. Move the arm gently and slowly during procedure. Avoid jerky movements.
4. The Arm should be gently put back into the socket after each procedure and in between subsequent passes.
5. Keep the treatment area clear for smooth movement of the arm.
6. The treating doctor has to first assess the most comfortable position to carry out the procedure for both the patient as well as the doctor without moving the hand piece very far away.
7. If treating large areas such as body, back or arms, the treating doctor should adjust the height according to their comfort either by putting a stool to sit or elevating or decreasing the height of the bed.
8. The hand piece lens has to be cleaned before and after every procedure especially after carbon peel laser toning (Q‐Laser) and ablative lasers where tissue splatter is expected.
9. Make sure no dust or hair particles have settled on the lens surface before lazing
10. Always use a smoke evacuator while using carbon peel or any ablative procedures
11. Do not fire the laser while the arm is hanging. It can damage the screen of laser and cause accidental injuries.

#### 2.4.3 Delivery of laser shots

1. While doing laser, hand piece should be in proximity toward lesion site and the laser beam to be perpendicular to the skin surface.
2. Hand movements to be swift and firm and in case of curved surface, the hand movements to be at the wrist level.
3. During procedure the operator and patient should not move suddenly as the laser shot may be delivered elsewhere.
4. The timing of foot switch should be synchronous with the laser shot.
5. Do not remove the foot from the foot switch in between the Laser shots. The foot should be over the foot switch with the heel on the ground and only the forefoot should move up and down for shots.
6. Foot should be moved away from foot switch once the procedure is done to prevent accidental firing while putting back the hand piece.
7. After laser procedure is done, make sure the foot switch is kept in a secured place to avoid tripping. Some laser devices come with a slot for foot switch placement behind the laser. One should be aware of the foot pedal wiring otherwise the operator may trip.
8. The wiring for foot pedal connections in most instances are flimsy hence secured with a duct tape.
9. Plan the treatment parameters fully before starting, particularly, when multiple different parameters are needed for the same patient (such as acne scars). Avoid putting the machine on standby mode, every time change in parameter is needed. This will strain the light source.
10. Adjust the frequency of laser shots as per convenience and comfort of the patient and operator.
11. Remember to clean the lens with isopropyl alcohol spray after every single use. Any debris should be removed from the lens with isopropyl alcohol and bud.

#### 2.4.4 Protective eye wear

1. Safety eyewear specific for Laser wavelength must be worn by operator, patient, and assistants in the room.
2. Recognize each type of goggles for each laser before putting it on, better to store them in boxes with wavelength specifications labeled on top of box.^14^, ^15^, ^16^
3. Handle the goggles with care and clean them with nonabrasive soft cloths.
4. Clean the glasses with saline swab before and after every use. Avoid using alcohol swab, unless contaminated with biological products, as it may corrode the coating on glasses.
5. Patient should always use protective goggle irrespective of area treated.
6. While treating inside the orbital rim intra‐corneal eye shields must be used.
7. Placement of corneal shield to be carried out under all sterile precautions.
8. Two drops of Paracaine 0.5% (w/v) ophthalmic solution are instilled in the treating eyes and waited for 1 min.
9. The corneal shield is held with the help of a sterile rubber suction gripper. One can also use corneal shields with metal handle for convenience.
10. Initially the lower eyelid is retracted and one end of the shield is tucked in gently while slowly releasing the eyelid. Then the upper eyelid is retracted and the other end of the shield is placed in.
11. Patient is asked to gently close their eyes and counseled that there will be pitch dark experience throughout the procedure and assured.
12. Once the procedure is completed, the shield is held with the help of the gripper and gently pulled out.
13. The corneal shield is washed, sterilized in autoclave, and kept wrapped in sterile cotton gauze for re‐use.
14. The plastic material corneal shields are preferable for procedures involving heat rather than stainless steel as the metal gets heated up and may damage the cornea. Example: Plastic corneal shields for Bipolar radio frequency used in skin tightening. Stainless steel corneal shield can be used for q switched, Pico lasers, fractional ablative lasers, long pulse Nd: yag, Alexandrite, Ruby laser etc.

#### 2.4.5 Sterilization of equipments

1. Probe and Machine—Wipe with Chlorhexidine swabs/alcohol swabs/spirit swabs followed by cleaning it with dry gauze.
2. Discard the disposable items, kidney tray to be autoclaved.
3. Glasses to be regularly cleaned with alcohol swabs before and after use.
4. Metallic patient eye cover to be sterilized after every procedure.
5. Disposable ice packs to be used in case of co2 lasers as it involves oozing of blood and serums. Avoid reusable ice packs.

### 2.5 Post procedure instructions to patients

1. Avoid active sunlight exposure at least for 1‐week post procedure and up to 2–3 weeks for resurfacing procedures.
2. Avoid face wash 6–8 h after procedure. To use a mild cleansing lotion to wash the treated area for 2–3 days post procedure.
3. To use a good sunscreen lotion/ cream/ gel in adequate quantity every 2–3 h for the treated area. Additional use of physical coverage is encouraged.
4. To use a good moisturizing cream or lotion in case of dryness or as a base cream at night.
5. To use a mild topical steroid cream once or twice daily for 3 days if there is burning sensation, burns or redness over the treated area as advised by treating physician.
6. Advise oral analgesics and oral steroids for short course in case of moderate to severe inflammation post procedure to minimize the risk of post inflammatory pigmentation.
7. Use of depigmenting creams/ actives to be started 4–10 days after the procedure at night (for resurfacing procedures with more downtime one can start after 7–10 days after skin heals).
8. Review after 2–3 weeks for next session in case of laser toning, 4–6 weeks in case of LHR and ablative lasers, 6–8 weeks in case of vascular lesions and tattoos.
9. Provide with an emergency contact number, email id to the patient in case of any adverse events or any doubts to be cleared.
10. Advise to strictly adhere to doctor's instructions and to contact office in case of unusual skin reaction or for any relevant doubts.

## 3 STANDARD OPERATIVE PROTOCOLS FOR SPECIFIC PROCEDURES

### 3.1 Laser hair reduction

#### 3.1.1 General instructions for hair reduction lasers

1. Avoid trimming of hair in the laser room area to avoid collection of unsterile matter. It has to be done in a room dedicated separately for patient preparation to avoid dust and hair settling on the lens.
2. Apply a cleansing gel prior to shaving the area to avoid cuts that may occur with dry shaving and to depilate in the direction of hair growth.
3. Ensure privacy while shaving.
4. If an electric shaver is used, the setting used should not leave residual hair growth more than 1 mm. Remove the struck hair pieces with plaster /cello‐tape.
5. Take history of medications (oral/topical) and rule out intake of any photosensitizing agents including but not limited to allopathic drugs, ayurvedic, homeopathic medications, home remedies.
6. Ask for any parlor procedure in the past 1–2 weeks including but not limited to sauna, steam bath, aromatic oil massages, scrubs, bleaching, etc. and for LHR procedures, hair removing parlor procedures like threading and waxing should be avoided 1 month prior to procedure.
7. Ask for any recent outdoor activities like hiking, trekking, beach visit etc. that could lead to tanning.
8. To rule out active/ previous episodes of herpes by examining and proper history taking in patient's own understandable language.
9. To cleanse the treatment area before lasing the area. In case the patient has applied makeup, cleansing the treatment area with micellar water is advised to aid complete removal of the tinted sunscreen, makeup etc.
10. Trim the hair in desired area, mark the areas using white marking pencil only with patient approval.
11. To discuss with the primary consultant regarding the machine and parameters/change in parameters.
12. The operator must mentally practice the procedure and rehearse.
13. Inspect the skin and be careful if any nevus/tattoo/abrasion/cut in the treatment area vicinity and cover them with micropore tape.
14. Cover eyebrow/hairline/side brows with tape.
15. Beard line, side burns to be marked and reconfirmed with the patient.
16. Shift the patient to the freshly prepared treatment chair.

#### 3.1.2 During the procedure

1. To use standard parameters as per protocol; use cryo air for skin cooling if needed (non‐contact lasers) and if there is not sufficient cooling from the laser device.
2. Hold the laser probe vertically and move perpendicularly.^17^, ^18^, ^19^
3. Move gradually with an appropriate overlap depending on the laser type used (contact vs. noncontact and Gaussian vs top hat beam). Check after few shots for any skip areas and the assistant must ensure the same.
4. For larger areas mark grids using a white marker (not colored) to avoid skip areas. Ensure that the borders are not skipped.
5. Perform the procedure smoothly, avoid jerks and finish the procedure in allotted time.
6. After completing one area, observe the area for any undue erythema and edema.
7. Body hair removal requires long durations, often leads to boredom/fatigue as the procedure is long. If so, exchange with another technician/doctor.
8. Engage the patient in conversation and play ambient music as per patient's choice.
9. Start with the least cosmetically visible areas first and look for tolerance of the patient, skin changes, adverse reactions; if tolerated (wait for 2 min) then move to other areas. Example: if treatment areas include Face and Neck; to start with neck then move to face. Start with the most lateral area then move medially.
10. Enquire about pain, discomfort, burning after first few passes/stamps.
11. Ensure proper lighting and positioning of the patient to have a proper look at the area being treated.
12. Warning signs in skin to be noted: whitish or grayish discoloration, dusky erythema, crumpled appearance of the skin.^20^
13. If a patient who has undergone multiple sessions, complains of pain at the same parameter, it needs to be taken as warning sign to check parameters again and take detailed history.
14. Stop the procedure if warning signs appear—erythema, burning, epidermolysis etc.^20^
15. After each body part, example—upper lip, left, right, cheek left, right—inspect the area, to look for warning signs before moving to next area.
16. If there are any warning signs, take an opinion from the senior doctor.
17. Lasing at the corner of the lips – ask the patient to press his/her tongue against inner surface or upper lip.

**Contact lasers (diode) or an IPL** ^17^, ^18^:

1. Consists of a contact hand pieces with sapphire tip for cooling.
2. Handle the tip with care to avoid any cracks in the tip.
3. Ensure adequate gel application for the treatment area for better gliding movements and better optical interphase.
4. Handpiece tip to be always in contact with skin during lasing to prevent burns.
5. During the procedure hand piece should be overlapped (20%–30%).
6. In case of any doubt after lasing, remove the gel and observe for signs of burns.
7. Post procedure, clean the tip with alcohol swab after removing gel.
8. Mild erythema may be noted as endpoint.

**Non‐contact hand pieces (Nd: YAG/ alexandrite lasers)** ^18^, ^19^:

1. Laser beam to be delivered in a perpendicular manner.
2. Hair should be trimmed before the procedure as the hair absorbs the energy and leads to inadequate energy delivery to the hair bulb and may also cause burns.
3. Cryo air to be used during the procedure to cool the epidermis.
4. Do an overlap more than 10%–20% of laser beam while lasing.
5. Hair singeing/peripilar edema/mild erythema may be noted as endpoint.

#### 3.1.3 After the procedure

##### Dos

1. To give adequate skin cooling immediately after procedure with cool air or ice packs.
2. To apply sunscreen and/or moisturizer and mild steroid cream in case of redness or burning.
3. A stat dose of oral analgesics and anti‐inflammatory to be given in case of burns and asked to continue it for 3 days.
4. In case of burns, colloid dressings and Silver nitrate ointments can be advised for 10–14 days.
5. Follow up the patient on day 1 and day 5.
6. If patient has any adverse effects, ask patient to visit—if they cannot, ask them to send photos and discuss with senior doctor.
7. Use remove sprays to remove the burnt hair odor.

##### Don'ts

1. Continuing the procedure with history of tanning, parlor procedure, photosensitivity and if herpes is present.
2. Any active viral, bacterial, or fungal infection at the site of treatment.
3. Starting the procedure in most visible areas.
4. Trying to finish the procedure in haste using higher repetition rates, skipping areas.
5. Ignoring warning signs from patient and skin reaction.
6. Any major upcoming event in the next 5 days.

### 3.2 Carbon dioxide laser

#### 3.2.1 Components of laser apparatus

Hand pieces—Fractional and surgical. The Laser arm is a joint articulated arm with changeable hand piece. It has 360‐degree rotation and must be handled delicately.

#### The fractional hand piece

Usually comes as scanner which allows to use different shapes and sizes depending on the treatment area. The available shapes are square, rectangle, triangle, and full/half circle.

Pitch distance: represents the distance between micro thermal zones (MTZ). By reducing the pitch distance, the fractional property is reduced and increasing the pitch distance results in increase in unaffected tissue area making the beam more fractional.

Stacking: refers to repetitive shooting at a particular spot, which results in increase depth of laser penetration while minimizing the damage to overlying epidermis, instead of using a higher fluence to achieve the same depth.

The laser beam dissipates energy in the form of a “V” shape or a conical shape in 3D, that is, more of epidermal damage and less of dermal damage.

#### Surgical handpiece

Comes with changeable spot sizes. It can be used in three modes: continuous wave, single pulse, and ultra pulse. The spot size must be set using the scale present on the hand piece.

**Continuous wave mode**: is operator dependent, as long as the foot switch is pressed the laser energy is delivered and hence depth achieved depends on the fluence used and the duration of application.

**Single pulse mode**: The laser energy is delivered in pulses with on and off time. It is used to precisely ablate desired tissue to a desired depth without causing damage to surrounding tissue. The power (W) can be modulated directly while the pulse duration as well the repetition rate will adjust to the given power automatically.

**Ultra pulse mode**: Utilizes pulse duration in terms of microseconds hence the beam causes less epidermal damage while penetrating, hence forms a ‘barrel shaped’ defect rather than “V” shape/ 3D cone.

- This reduces the untoward effects of laser beam interaction with the epidermis and desired fluence/power can be used to treat a well delineated lesion.
- Shouldering, ablating benign lesions to be used in ultra‐pulse mode/single pulse mode.

Surgical hand pieces are usually zoom hand pieces and if placed close to skin surface they provide more cutting and if slowly moved from treatment surface can add coagulative effect for the same parameters.

#### 3.2.2 General instructions before Co2 laser

1. Shift the patient on the freshly prepared treatment couch, drape, and respect patient's privacy
2. Remove the topical anesthesia with spatula
3. Clean the skin with betadine/ saline. Avoid alcohol swabs as it is inflammable
4. Topical anesthesia or non‐contact cooling by cold refrigerated air (example zimmer) is provided to the treating area during the procedure.
5. If patient is allergic to topical anesthesia or the level of treatment area is deep—nerve block to be given under asepsis.
6. Head band and eye guard to be worn.
7. Explain the patient regarding the procedure steps. Counsel regarding minimal pain/ heat and discomfort during the procedure.
8. Switch on the machine and set the parameters.
9. Recheck the machine parameters.

#### 3.2.3 During the procedure

1. When delivering the shot, the fractional hand piece should be placed perpendicular to the skin surface.
2. Inspect, look for uniformity after the procedure, and ensure that there are no skip areas.
3. Wherever necessary feathering of scars edges to be done to prevent color contrast post healing.
4. Surgical hand piece is used for shouldering of scars and to treat benign skin lesions.^21^, ^22^
5. For treating benign skin lesions the surgical hand piece is held straight and perpendicular to the treatment area. Choose the appropriate spot size and pulse duration depending on the treatment lesion. One can use Pulse mode, for better control or continuous wave mode. Lase the benign lesion first and later level it up to the surrounding skin. A slight pink color end point is desirable.
6. While performing the shouldering for atrophic scars the hand piece is to be held at an acute angle targeting the edge of the scars Use ultra‐pulse/Pulse mode.^21^, ^22^
7. Cool the area with ice packs for 15 min till the patient feels little or no burning.

#### 3.2.4 After the procedure

1. Apply moisturizer, steroid and sunscreen immediately after procedure.
2. Motivate the patient to follow strict sun protection/sun avoidance.
3. If needed a colloidal dressing can be applied on treated area to help for faster recovery.
4. Application of steroids for 5–7 days following procedure if severe edema is anticipated.
5. Oral analgesics in case of mild to moderate pain.
6. Follow up the patient the Day 1 and Day 7 for enquiry about any side effects and recovery.

The Depigmenting creams and face washes can be resumed after a 7–10 days.

### 3.3 Qs—nd yag laser (pico/nano—laser toning/tattoo)^23^, ^24^, ^25^

#### 3.3.1 General instructions for q‐switched lasers

1. Consent, photo documentation and brief counseling on procedural steps to be done.
2. Cleanse/wash the area to be treated to remove make up.
3. The Doctor must check the lesion type and skin before applying EMLA.
4. Apply topical anesthesia cream when indicated and the cream needs to be removed after 30–45 min of application.
5. Shift the patient on the freshly prepared treatment couch once ready for treatment.
6. Remove the EMLA with spatula and cleanse the area.
7. Make sure the room is adequately air conditioned, with no mirrors in the room and doors are closed.
8. Check the availability of smoke evacuator especially for carbon peel.
9. Check if the machine is placed in comfortable position to perform the procedure.
10. The treatment parameters for each lesion should be set as per the Doctor's written instructions—energy, power, pulse width and spot size.
11. Before each treatment procedure, recheck the parameters and test fire the laser system on black paper to confirm that it is working optimally.
12. Give drapes/ sheets to cover the rest of the areas where indicated.
13. Adequate eye protection should be worn by all the people in the procedure room strictly.

#### 3.3.2 During the procedure

1. Explain patient about mild or minimal pain / sounds experienced.
2. Turn the key to switch on the machine; some machines may have a password. Allow the machine to warm up.
3. Set the parameters as advised by consultant and set the probe and mode as desired.
4. Make sure the smoke evacuator is switched on while performing carbon peel step.
5. A test shot must be performed in a small 1 × 1 cm area before proceeding with the treatment; the end point being visualized clinically and by a dermoscopy before proceeding over full face.
6. The operator should always handle the hand piece in a safe manner to avoid accidental firing.
7. The hand piece tip should always be positioned perpendicular to the skin surface.
8. Press hand piece firmly with mild traction with slight pressure.
9. After each use, the hand piece should be placed back into the hook located on the system's arm.
10. Only the operator should control the foot pedal during the treatment procedure.
11. For lesions near the eyes, extra precautions to be taken in sensitive patients; use metal eye covers like steel spoons. While doing around the eyes intra‐corneal shield to be put.
12. Watch for the end point:
  - Tattoo removal: white frosting
  - Laser toning: Mild erythema/graying of hair/edema
13. Special precautions while performing Q‐switched lasers:
  - In case of freckles, precise targeting needs to be done. Each lesion needs to be checked to make sure the fluence is enough.
  - In case of tattoo removal—for a narrow tattoo fix a smaller spot size and higher fluence, for a wider tattoo set a larger spot size and lower fluence, as the tattoo fades, reduce the spot size.
  - In case of pin point bleeding and severe edema at test shot, −always lower the fluence, provide cooling to the area and then proceed.
14. Laser eye glasses may not allow precise determination of the frosting or mild edema; hence it is important to frequently inspect with naked eye during procedure.
15. The assistant should note the skin reaction after the shots periodically and give feedback.
16. If the patient is not comfortable, immediately stop the procedure and inform the Doctor.

#### 3.3.3 After the procedure

1. Machine to be turned off after the procedure.
2. Adequate cooling with ice packs to be given for 5–10 min. The ice compressions must be gentle for 3–5 s at site, done five times; do not put press nor keep compression for more than 5 s per site, to prevent cold injury.
3. Apply the moisturizer/ sunscreen gently, do not massage.
4. Look for any hot spots if undue erythema or edema before moving the patient.
5. Confirm that the patient has received the post procedure instructions.

### 3.4 Vascular laser^26^

#### 3.4.1 General instructions for vascular lasers

1. Patient consents, photo documentation should be done.
2. Topical EMLA to be applied for 35–40 min.
3. Remove the EMLA and cleanse the area to be treated
4. Mark the test spot area.
5. Eye gear to be worn by all the personal in the laser room.

#### 3.4.2 During the procedure

1. Turn on the machine.
2. Set the parameters, and then test shot must be given.
3. Test shots with different energies with varying pulse durations done on the treatment lesion. Area to be selected to be lateral or a hidden area.
4. Ideal end point for the test shot: Purpura to be visualized by a dermatoscope (Ash gray discolouration). Also note the broken vessels in the treatment zone.
5. Watch out for epidermolysis, severe purpura, burns. If yes reduce the energy.^26^
6. Once the energy is finalized, draw grids of 1 cm each across the treatment area and proceed with 1 pass in static mode.
7. Avoid using pre and perioperative cooling with icepacks or non contact cooling device as the vasoconstriction can interfere with laser interaction with hemoglobin, the target chromophore.

#### 3.4.3 After the procedure

1. Once the entire treatment area is treated, ice pack must be given to the patient.
2. Post procedure purplish discolouration of the treated area is expected and patient must be counseled regarding the same.
3. Apply topical steroid and sunscreen before seeing off the patient.
4. Advise patients to use protective physical covering in addition to the sunscreen.

### 3.5 Fractional microneedle radiofrequency MnRF^27^, ^28^

#### 3.5.1 Machine descriptions

Needle cartridge Tip: usually 4 different needles are available

1. Resurfacing tip with depth of 0.5 mm for skin rejuvenation.
2. 12 pin needles for treating periorbital and perioral areas
3. 24 pin needles, most used with varying depths up to 4 mm used for scars.
4. 40 pin needles for depths up to 7 mm, used for stretch marks company and cellulite.

##### Energy

1. Select optimum energy as per indication and area of treatment.
2. Note: as we come closer to skin surface use lesser energies to minimize epidermal damage.
3. MODE:
4. Fixed: the needles are fixed at a particular depth and you can stack the pulses.
5. Cycle: one pulse is delivered for 1‐foot pedal application. This mode is used when you do not need stacking of pulses. For example, on bony areas, nose, temple, periocular, perioral.
6. Burst mode: useful for treating stretch marks/ cellulite/ hiddradenitis suppartiva in pubic area/ keloid/hypertrophic scars.
  - In this mode same energy is delivered at different depths.
  - For example, 30 J 4 mm tip deliver 30 J at 4, 2, 1 mm

##### Repetition

1. Indicate the pulse delivered per second. Purely indicates the speed of the operator.
2. 0.5 PPS means for every 2 s one pulse is delivered
3. 1 PPS means 1 pulse per second and so on.
4. For beginners, try to use 0.5 PPS till one gets used to handling of handpiece.

#### 3.5.2 General instructions for MNRF procedure

1. Good quality photographs should be taken with appropriate background and lighting.
2. For a repeat procedure patient, check whether the patient has brought the MNRF cartridge. A patient can ideally use the cartridge for four to six sessions depending on the indication.
3. Assess the scars of the patient prior to application of topical EMLA.
4. Apply and leave an even coat of EMLA over the treatment areas for a period of at least 45 min.
5. Sterilize the area with betadine before starting. Leave the betadine for 2 min to ensure the treatment area is adequately clean. Clean the area with saline before starting the procedure.

#### 3.5.3 During the procedure

1. Ensure that the cartridge is inserted correctly in the hand piece. Set the parameters of the machine as advised by the consultant
2. Always make sure the needle is inserted completely before firing a shot. Insert the needle perpendicular to skin surface.
3. You can use Stretch / pinch technique for inserting needle.
  - Stretch technique: stretch the skin and then insert the needle. This is useful for even penetration of needles.
  - Pinch technique: Pinch the treatment area and then insert the needle. It is useful in areas like cheeks and has advantage of minimizing the pain for the patient.
4. Over curved areas, place a gauze piece over areas where pins are not in even contact with the skin to avoid epidermal burns.
5. Ensure an overlap of 30%–40% of hand piece to avoid skip areas considering the width of as the hand piece rim.
6. Decrease the fluence in bony areas.
7. While giving multiple passes, follow the same pattern and same starting point. The passes can be given in the following way; horizontal, then vertical, and finally diagonal directions.
8. First remove the foot from the foot pedal and then slowly remove the hand piece from the one treatment area before inserting into another area. This prevents accidental firing of shot while removing the hand piece and thereby epidermal injury.
9. In procedures where PRP is combined, spray the PRP on the MnRF treated area and massage it slightly. Some amount of PRP can be injected for deep scars. Doctor should change the gloves to surgical gloves before applying PRP.
10. Beyond 3 mm, bi‐level anesthesia is required.
11. If patient unable to tolerate pain, pinch the skin upward prior to delivering the shot (Pinch technique).

#### 3.5.4 After the procedure

On completion of procedure ensure that cartridge pin is sanitized adequately using alcohol. Handover the cartridge to the patient in a cover and ask the patient to bring it back every time when they come for procedure.

1. Post procedure: Ask the patient to apply Ice packs for a duration of 15 min.
2. Advice the patient that erythema and edema may persist for up to 3 to 5 days.
3. Apply a thick layer of sunscreen before sending the patient home.
4. Advise sunscreens during daytime, moisturizers at night and cleansers for face wash (for first 5 days).
5. After 1 week a skin lightening cream with Kojic acid should be advised twice daily on the treatment area for 2 weeks.
6. A Skin repair gel can be advised for first 1‐week post procedure.

### 3.6 Cryolipolysis^29^, ^30^, ^31^

#### 3.6.1 General instructions before cryolipolysis procedure

1. Confirm areas on the body patient is wanting to treat.
2. Provide the patient review consent forms and confirm there are no contraindications present. such as Reynaud's, cold‐sensitive disorders, hernia, or mesh in the treatment area.
3. Inform the patient of swelling, bruising, numbness that can occur post treatment.
4. Give patient disposable underwear and instruct to stand in designated photo area.
5. Photos taken of the area being treated while standing on a photo wheel to ensure all photos will be taken at the same angles.

#### 3.6.2 During procedure

1. Assess the treatment area by pinching the fat at eye level to confirm area has subcutaneous fat that can be treated and then initiate marking.
2. Locate with hands where fatty areas are most prominent and mark them with an X.
3. Use the measuring cups provided to ensure there is enough subcutaneous tissue to treat, trace desired applicator placement onto area and make lines where needed to indicate appropriate angles needed.
4. Copy all markings on the treatment record form so that a visual aid is available for provider and patient regarding exact placement of each applicator.
5. Document on treatment record form which size and shape applicator will be used to ensure future sessions will be followed up accurately.
6. After desired pattern is created and patient is satisfied with plan, instruct patient to get onto treatment table in appropriate position dependent on area.
7. Insert appropriate applicators into hoses on device and enter in patient's information such as sex, if it is their first treatment, phone number for tracking rewards, and how many cycles they will be receiving during session.
8. Clean the skin with pre‐treatment skin wipes thoroughly for 60 s over each application marking.
9. Apply gel pad directly on top of applicator marking.
10. Turn on suction of applicator and guide directly on top of applicator marking made until it makes full contact.
11. Using straps and Velcro provided, wrap the applicator around the area being treated and secure tightly using pillows or towels to stabilize.
12. Check applicator by listening for any air escaping or “bubbling” and adjust as needed.
13. Once applicators are secure and patient is comfortable, follow checklist prompts on device to confirm all steps were taken for application.
14. If utilizing a second applicator to run 2 cycles simultaneously, clean and apply gel pad with applicator to second area being treated after confirming it has no conflict with first applicator placement such as tugging skin that would break suction contact.
15. Wrap second applicator tightly and secure, and then follow prompts to confirm all steps were taken for application.
16. Press START on first treatment cycle, and wait 5 min before pressing start on second to make time for post treatment massage.

#### 3.6.3 After procedure

1. Once first treatment cycle is complete, turn off the suction and massage area for 2 min immediately after removing the applicator. Begin massage on second area immediately post treatment as well.
2. Clean the area off with a wet, warm towel to ensure all the gel has been removed from the skin.
3. Encourage patient to use compression garments to help with swelling and to stay hydrated, and to avoid extreme hot or cold temperatures.
4. Make follow up appointment 1–3 months out to continue treatment plan and document progress with more photos.

## 4 CONCLUSION

The above protocols while performing the energy‐based devices helps to maintain the uniformity and avoid complications. However, these instructions are generalized and not machine or lesion specific. There may be variations in the protocols depending on the treatment lesion and treating doctor as well as machine.

## AUTHOR CONTRIBUTIONS

I agree that all the above‐mentioned authors have participated and contributed for the current article.

## ETHICAL STATEMENT

Not applicable.

## Data Availability

Data sharing is not applicable to this article as no new data were created or analyzed in this study.

## References

[1] King M . Prophylaxis and Treatment of Herpetic Infections. J Clin Aesthet Dermatol. 2017;10(1):E5‐E7. PMC5300736 28210384

[2] Nestor MS . Prophylaxis for and treatment of uncomplicated skin and skin structure infections in laser and cosmetic surgery. J Drugs Dermatol. 2005;4(6):s20‐s25. 16300226

[3] Koay J , Orengo I . Application of local anesthetics in dermatologic surgery. Dermatologic Surg. 2002;28:143‐148. 10.1046/j.1524-4725.2002.01126.x 11860425

[4] Epstein RH , Halmi B , Lask GP . Anesthesia for cutaneous laser therapy. Clin Dermatol. 1995;13:21‐24. 7704851 10.1016/0738-081x(95)92695-d

[5] Drake LA , Dinehart SM , Goltz RW , et al. Guidelines of care for local and regional anesthesia in cutaneous surgery. J Am Acad Dermatol. 1995;33:504‐509. 7657874 10.1016/0190-9622(95)91397-1

[6] Dhepe N . Minimum standard guidelines of care on requirements for setting up a laser room. Indian J Dermatol Venereol Leprol. 2009;75:101‐110.

[7] Ashique KT , Kaliyadan F , Aurangabadkar SJ . Clinical photography in dermatology using smartphones: an overview. Indian. Dermatol Online J. 2015;6(3):158‐163. doi:10.4103/2229-5178.156381 PMC4439742 26009708

[8] Kaliyadan F , Manoj J , Venkitakrishnan S , Dharmaratnam AD . Basic digital photography in dermatology. Indian J Dermatol Venereol Leprol. 2008;74:532‐536. 19052435 10.4103/0378-6323.44334

[9] Katoch S , Mysore V . Surgical smoke in dermatology: its hazards and management. J Cutan Aesthet Surg. 2019;12(1):1‐7. doi:10.4103/JCAS.JCAS_177_18 31057262 PMC6484569

[10] Das A , Sarda A , De A . Cooling devices in laser therapy. J Cutan Aesthet Surg. 2016;9(4):215‐219. doi:10.4103/0974-2077.197028 28163450 PMC5227072

[11] El Arabi Y , Hali F , Chiheb S . Laser management and safety in dermatology. Cureus. 2022;14(6):e25991. doi:10.7759/cureus.25991 35859982 PMC9287998

[12] Steen WM . Laser Safety. Laser Material Processing. Springer; 2003. doi:10.1007/978-1-4471-3752-8_12

[13] Drake LA , Ceilley RI , Cornelison RL , Dinehart SM , Dorner W . Standards of training for physicians for the use of lasers in medicine and surgery, American Society for Laser Medicine and Surgery. Inc J Am Acad Dermatol. 1995;33:265‐270. 10.1016/0190-9622(95)90260-0 7622655

[14] Russell SW , Dinehart SM , Davis I , Flock ST . Efficacy of corneal eye shields in protecting patients' eyes from laser irradiation. Dermatologic Surg. 1996;22:613‐616. 10.1111/j.1524-4725.1996.tb00607.x 8680783

[15] Malayanur D , Mysore VN . Laser safety eyewear. CosmoDerma. 2022;2:24.

[16] Wamsley CE , Hoopman J , Kenkel JM . Safety guidelines concerning the use of protective eyewear and gauze during laser procedures. Aesthet Surg J. 2021;41(10):1179‐1185. doi:10.1093/asj/sjaa233 32756948

[17] Buddhadev RM . Standard guidelines of care: laser and IPL hair reduction. Indian J Dermatol Venereol Leprol. 2008;74:68‐74. 18688107

[18] Casey A , Goldberg D . Guidelines for laser hair removal. J Cosmet Laser Ther. 2008;10:24‐33. doi:10.1080/14764170701817049 18330795

[19] Liew SH . Laser hair removal: guidelines for management. Am J Clin Dermatol. 2002;3(2):10715. doi:10.2165/00128071-200203020-00004 11893222

[20] Hussain M , Polnikorn N , Goldberg DJ . Laser‐assisted hair removal in Asian skin: efficacy, complications, and the effect of single versus multiple treatments. Dermatologic Surg. 2003;1:249‐254. 10.1046/j.1524-4725.2003.29059.x 12614418

[21] Krupashankar DS . Standard guidelines of care: CO _2_ laser for removal of benign skin lesions and resurfacing. Indian J Dermatol Venereol Leprol. 2008;74:61‐67. 18688106

[22] Krupa Shankar DS , Chakravarthi M , Rachana S . Carbon dioxide laser guidelines. J Cutan Aesthet Surg. 2009;2(2):72‐80. doi:10.4103/0974-2077.58519 20808594 PMC2918344

[23] Shah SD , Aurangabadkar SJ . Laser Toning in Melasma. J Cutan Aesthet Surg. 2019;12(2):76‐84. doi:10.4103/JCAS.JCAS_179_18 31413475 PMC6676813

[24] Sardana K , Ranjan R , Ghunawat S . Optimising laser tattoo removal. J Cutan Aesthet Surg. 2015;8(1):16‐24. doi:10.4103/0974-2077.155068 25949018 PMC4411587

[25] Chandrashekar BS , Shenoy C , Madura C . Complications of laser and light‐based devices therapy in patients with skin of color. Indian J Dermatol Venereol Leprol. 2019;85:24‐31. 30560814 10.4103/ijdvl.IJDVL_88_17

[26] Srinivas CR , Kumaresan M . Lasers for vascular lesions: standard guidelines of care. Indian J Dermatol Venereol Leprol. 2011;77:349‐368. 21508585 10.4103/0378-6323.79728

[27] Chandrashekar BS , Sriram R , Mysore R , Bhaskar S , Shetty A . Evaluation of microneedling fractional radiofrequency device for treatment of acne scars. J Cutan Aesthet Surg. 2014;7(2):93‐97. doi:10.4103/0974-2077.138328 25136209 PMC4134659

[28] Tan MG , Jo CE , Chapas A , Khetarpal S , Dover JS . Radiofrequency microneedling: a comprehensive and critical review. Dermatologic Surg. 2021;47(6):755‐761. 10.1097/DSS.0000000000002972 33577211

[29] Gold M , Kleinrock M , Utley C , Bauknecht C , Goldberg D . Nonsurgical fat reduction: Cryolipolysis, RF skin tightening, HIFEM, and injectable lipolysis. Dermatol Rev. 2021;2:196‐204. doi:10.1002/der2.79

[30] Few J , Saltz R , Beaty M , et al. Cryolipolysis: clinical best practices and other nonclinical considerations. ASJ Open Forum. 2020;2(2):ojaa010. 33791637 10.1093/asjof/ojaa010 PMC7671251

[31] Ali T , Habib A , Riaz R , Shaukat A , Akilimali A . Assessing the benefits and drawbacks of cryolipolysis as a noninvasive body sculpting technique: risks, side effects, and future research–an editorial. IJS Glob Health. 2023;6(5):e0340.

